# Effects of Partial Myostatin Loss of Function on Carcass Yield and Meat Quality in Pigs Differing in IGF2 Genotype

**DOI:** 10.3390/ani16172634

**Published:** 2026-08-22

**Authors:** Elli S. Burris, Lauren T. Honegger, Dustin D. Boler, Jonathan E. Beever, Anna C. Dilger

**Affiliations:** 1 Department of Animal Sciences, University of Illinois, Urbana, IL 61801, USA; ellisj2@illinois.edu (E.S.B.); dboler2@illinois.edu (D.D.B.); 2 Department of Animal Science, University of Tennessee, Knoxville, TN 37996, USA; jbeever@utk.edu

**Keywords:** belly quality, carcass composition, gene editing, IGF2-G3072A, lean yield, MSTN, pork quality, tissue partitioning

## Abstract

The objective of this study was to evaluate the effects of partial loss of function (LOF) of MSTN on carcass composition and pork quality and determine whether the phenotype was influenced by the IGF2 genotype (IGF2-G3072A). Pigs (N = 44) segregating for the paternally expressed IGF2 G or A alleles were either wild-type (WT) or heterozygous (HET) for a targeted MSTN LOF allele and were harvested at 175 ± 5 days of age. Carcass composition and loin and belly quality traits were analyzed using linear models. Few significant MSTN × IGF2 genotype interactions were detected, indicating that the heterozygous MSTN phenotype was largely independent of IGF2 status. Relative to WT contemporaries, MSTN HET pigs exhibited greater dressing percentage and loin muscle area (*p* < 0.01) without differences in hot carcass weight or backfat depth, resulting in increased fat-free lean percentage. However, loins from HET pigs were approximately 5 L* units lighter in color (*p* < 0.01) and exhibited greater drip loss. Marbling scores and extractable lipid content were reduced in both loin and belly, and belly firmness decreased. The IGF2 genotype had similar muscle-deposition effects. These results indicate that partial MSTN LOF improves lean yield but introduces tradeoffs in pork quality, largely independent of IGF2 genotype.

## Simple Summary

Improving lean meat production without reducing pork quality is an important goal for the swine industry. A gene called myostatin naturally limits muscle growth in animals. In this study, pigs carrying one reduced-function copy of the myostatin gene produced more lean meat and larger loin muscles than pigs with normal myostatin. However, these improvements were accompanied by some undesirable changes in meat quality, including lighter-colored loins with greater moisture loss and thinner, softer bellies with less fat. These effects were similar regardless of which version of another growth-related gene, *IGF2*, pigs carried. Overall, partially reducing myostatin function increased lean meat yield but also created tradeoffs in meat quality that should be considered before using this approach in commercial pork production.

## 1. Introduction

Myostatin, encoded by MSTN, is a member of the transforming growth factor-β (TGF-β) superfamily and functions as a potent negative regulator of skeletal muscle development, limiting both prenatal myoblast proliferation and postnatal muscle hypertrophy [1]. Naturally occurring loss-of-function (LOF) mutations in MSTN have been identified in cattle, sheep, mice, dogs, and humans and are associated with increased muscle mass and the well-characterized “double-muscling” phenotype [2,3,4,5,6]. In contrast, no spontaneous MSTN LOF allele has become widely established in commercial pig populations. A naturally occurring premature stop mutation in MSTN has been identified in a commercial line; however, it is associated with a recessive leg weakness syndrome and reduced survival of homozygous animals, despite evidence of increased muscle depth in heterozygotes [7]. These findings suggest that complete MSTN deficiency may be selectively constrained in pigs.

Beyond their effects on muscle mass, MSTN LOF mutations have also been associated with changes in meat quality. Across livestock species, reduced MSTN activity has been linked to decreased fat deposition and intramuscular fat content, whereas the effects on postmortem quality traits such as tenderness, water-holding capacity, and color have been less consistent and appear to depend on species, genetic background, and the extent of myostatin inhibition [2,8,9,10,11]. Consequently, while the effects of MSTN on carcass composition are well established, its influence on pork quality traits remains less clearly defined.

Gene-edited pigs with disrupted MSTN have also been generated; however, outcomes have varied by genetic background. Early reports in commercial-type breeds demonstrated that pigs homozygous for engineered MSTN LOF alleles exhibited severe mobility impairment and high postnatal mortality [12,13]. Similar phenotypes were observed regardless of whether CRISPR/Cas9 or TALEN-based editing approaches were used, indicating that lethality was attributable to MSTN deficiency rather than the editing platform. In contrast, viable MSTN-null animals have been reported in Erhualian pigs, a Chinese indigenous breed with a genetic background distinct from intensively selected commercial lines [14,15]. These contrasting outcomes raise the possibility that genetic background, rather than MSTN deficiency, influences postnatal viability in pigs.

One major genetic distinction between many commercial pig populations and certain unselected or indigenous breeds is the presence of a regulatory mutation in intron 3 of IGF2 (IGF2-G3072A). This variant disrupts binding of the transcriptional repressor ZBED6, resulting in increased postnatal skeletal muscle expression of IGF2 [16]. Because IGF2 is maternally imprinted in pigs, only the paternal allele is expressed in skeletal muscle; consequently, the IGF2-G3072A variant affects growth only when it is inherited from the sire. Intensive selection for lean yield has increased the population frequency of the A allele in commercial swine, establishing a genetic background characterized by widespread elevated IGF2 expression. The paternal A allele is associated with increased loin muscle area, reduced backfat depth, lighter loin color, and altered belly quality [17,18]. Thus, long-term selection for lean growth in commercial pigs has largely occurred in the context of enhanced IGF2 signaling.

Evidence from murine models suggests functional overlap between MSTN and IGF2 signaling pathways. Mice lacking MSTN exhibit increased expression of *Igf2* during postnatal development, indicating a potential interaction between these growth-regulatory networks [19]. Given that most commercial pig populations have a high frequency of the IGF2 A allele, additional disruption of MSTN may produce attenuated effects on muscle accretion or introduce deleterious epistatic interactions.

To evaluate the influence of the IGF2 genotype on the phenotypic consequences of MSTN deficiency, a targeted LOF mutation was introduced into exon 3 of MSTN using zinc-finger nucleases in fetal fibroblasts heterozygous for IGF2 [20]. Pigs were generated via somatic cell nuclear transfer and subsequently interbred to produce animals differing in paternal IGF2 genotype and MSTN status. Consistent with previous reports, pigs homozygous for the MSTN LOF allele exhibited low postnatal viability, whereas heterozygous animals were viable.

In several livestock species, heterozygous MSTN LOF genotypes confer increased muscle mass without the extreme phenotype observed in homozygous individuals [21,22]. Therefore, the objectives of this study were to characterize the effects of partial MSTN loss of function on carcass composition and meat quality in pigs and to determine whether these effects are also influenced by the paternal IGF2 allele. We hypothesized that the phenotypic response to heterozygous MSTN deficiency would differ between pigs inheriting the paternal IGF2 G allele and those inheriting the A allele, reflecting an interaction between these two growth-regulatory pathways.

## 2. Materials and Methods

Prior to experimentation, the University of Illinois Institutional Animal Care and Use Committee approved the experimental protocol for this study.

### 2.1. Generation of Myostatin Mutant Animals

A loss-of-function (LOF) allele of MSTN was generated using zinc-finger nucleases, as previously described [20]. Briefly, exon 3, which encodes the mature protein of *MSTN*, was targeted, resulting in a single-base-pair deletion (−1T) through non-homologous end-joining repair and a predicted frameshift mutation. Female embryos were produced via somatic cell nuclear transfer using edited fetal fibroblast cell lines carrying the −1T allele. Founder females were subsequently bred to commercial boars to expand the population. Through successive generations, the edited MSTN allele was maintained, and the population was managed for segregation of both alleles at the IGF2-G3072A locus. Genotypes at the MSTN locus were classified as wild-type (WT) or heterozygous (HET) for the edited −1T allele. Because IGF2 is maternally imprinted in pigs, experimental groups were defined by the paternally inherited allele (A^PAT^ or G^PAT^).

### 2.2. Experimental Animals and Design

A total of 44 pigs distributed across three farrowing blocks were included in the study. Block 1 consisted of 19 pigs produced from matings between commercial Yorkshire sows and a boar of mixed Berkshire and Yorkshire ancestry heterozygous for MSTN and heterozygous at the IGF2-G3072A locus (i.e., A/G). Blocks 2 and 3 comprised 25 pigs generated from matings between sows of mixed Berkshire and Yorkshire ancestry heterozygous for MSTN and a commercial Berkshire boar that was wild-type for MSTN and heterozygous for the IGF2 locus. Blocks 2 and 3 represent pigs farrowed at two separate time points.

Approximately 24 h after birth, piglets were weighed, ear-notched for identification, and processed according to standard industry practices, which included iron supplementation, tail docking, and surgical castration of males [23]. Ear tissue samples were collected for genotyping, as described by Clark et al. [19]. Genotypes were determined prior to weaning. Animals were classified into four groups based on the paternal IGF2 allele and MSTN status: G^PAT^ WT (n = 13), G^PAT^ HET (n = 10), A^PAT^ WT (n = 11), and A^PAT^ HET (n = 10).

Pigs were housed at the University of Illinois Imported Swine Research Laboratory and fed diets formulated to meet or exceed NRC [24] nutrient requirements for each production phase. Feed and water were provided ad libitum. Animals were housed in mixed-sex pens containing 9–15 pigs per pen. Genotypes were commingled within pens to minimize pen effects and to ensure that animals of each genotype were exposed to similar housing and feeding conditions. Because genotypes were commingled within pens, feed intake and growth rate were not recorded.

### 2.3. Slaughter and Carcass Quality

At 175 ± 5 days of age, pigs were transported to the University of Illinois Meat Science Laboratory (Urbana, IL, USA) for harvest. Upon arrival, animals were held in lairage for approximately 16 h with ad libitum access to water but no access to feed. Ending live weight (ELW) was recorded immediately prior to humane slaughter. Pigs were rendered unconscious using head-to-heart electrical stunning and subsequently exsanguinated under supervision of the United States Department of Agriculture Food Safety and Inspection Service (USDA-FSIS).

Carcasses were weighed approximately 45 min postmortem to determine hot carcass weight (HCW). Dressing percentage (%) was calculated as (HCW/ELW) × 100. Carcasses were chilled at 4 °C for approximately 20 h postmortem before the right side of each carcass was used for carcass composition measurements. Carcasses were ribbed between the 10th and 11th ribs to expose the longissimus thoracis et lumborum (LTL). Backfat depth was measured at this location. Loin muscle area (LMA) was determined by tracing the surface of the LTL onto acetate sheets. Tracings were digitized and analyzed by a trained technician using a digitizing tablet (Wacom, Vancouver, WA, USA) and Adobe Photoshop CS6 (Adobe Systems Inc., San Jose, CA, USA). The average of two measurements was used to represent LMA. Fat-free lean percentage (FFL, %) was calculated according to procedure 1 for ribbed carcasses [25] using the following equation: FFL (%) = [(8.588 + (0.465 × HCW, lb) − (21.896 × fat thickness, in) + (3.005 × LMA, in^2^))/HCW, lb] × 100.

### 2.4. Carcass Fabrication

At 1 d postmortem, chilled left sides were weighed. Leaf fat and visceral fat were removed and weighed separately. Adjusted chilled side weight was calculated as the hot carcass weight (HCW) minus the internal fat weight. Carcasses were fabricated according to specifications in the North American Meat Institute Meat Buyer’s Guide [26] into the following primals: pork leg (NAMP #401), skin-on whole loin (NAMP #410), pork shoulder (NAMP #403), skin-on natural fall belly (NAMP #408), and spareribs (NAMP #416). Each primal was weighed prior to further fabrication. Hams (NAMP #401) were skinned and trimmed to produce NAMP #402 hams and reweighed. The semitendinosus (ST) muscle was removed and weighed. Loins (NAMP #410) were skinned and further fabricated to yield the longissimus dorsi (Canadian back loin, NAMP #414) and psoas major (tenderloin, NAMP #415A). Shoulders (NAMP #403) were skinned and separated into bone-in Boston butt (NAMP #406) and bone-in picnic (NAMP #405). The triceps brachii (shoulder cushion) was removed from the picnic and weighed. Whole bellies with spareribs were weighed and subsequently fabricated into NAMP #409 bellies and reweighed.

### 2.5. Loin Quality Evaluation

Loin quality evaluations were conducted on the Canadian back loin (NAMP #414) by trained University of Illinois personnel on the day of fabrication (1 d postmortem). Measurements included ultimate pH, visual marbling, visual color, subjective firmness, and instrumental color and were collected in a refrigerated room. Loins were cut at approximately the 10th rib to expose the chop surface and were allowed to bloom for a minimum of 30 min prior to evaluation. Ultimate pH was measured using a handheld pH meter equipped with a glass electrode (MPI pH-Meter, Topeka, KS, USA) calibrated at pH 4.0 and 7.0. Instrumental color (CIE L*, a*, b*) was measured using a CR-400 Chroma Meter (Konica Minolta Camera Co., Ltd., Osaka, Japan) with a D65 illuminant, 10° observer angle, and 8 mm aperture. The instrument was calibrated using a standardized white tile prior to measurements. Visual color and marbling were evaluated according to NPPC standards [27], and subjective firmness was assessed following NPPC guidelines [28]. All subjective scores were assigned in whole or half increments by a single trained technician.

Following quality evaluation, a 0.64 cm-thick chop was collected for drip loss determination. Chops were weighed, suspended in individual plastic bags (Whirl-Pak; Nasco Sampling, Madison, WI, USA) to prevent surface contact, and stored at 4 °C. After 24 h, chops were removed, gently blotted dry, and reweighed. Drip loss was expressed as the percentage weight loss relative to the initial weight. Two additional 2.54 cm-thick chops were collected for proximate composition analysis and Warner–Bratzler shear force (WBSF) determination. Proximate composition samples were immediately frozen while WBSF samples were aged at 4 °C for 14 d prior to freezing. Samples were vacuum packaged and stored at −20 °C until analysis.

### 2.6. Belly Quality Evaluation

Fresh bellies were evaluated 1 d post-fabrication for length, width, thickness, and belly flop distance. Belly length was measured along the anterior-to-posterior midline, and width was measured at the widest dorsal-to-ventral point. Belly thickness was calculated as the average of eight measurements collected at 20%, 40%, 60%, and 80% of the total belly length on both the dorsal and ventral sides (four measurements per side). Belly flop distance was determined by placing each belly skin-side down over a standardized metal bar and measuring the linear distance between the anterior and posterior ends. Belly flop distance was used as an objective indicator of belly firmness, which is directly influenced by adipose tissue deposition. Following physical measurements, whole bellies were skinned and homogenized using a Talsa bowl chopper (C40P; Talsabell S.A., Valencia, Spain). Subsamples were collected, placed in Whirl-Pak bags (Nasco Sampling, Madison, WI, USA), and stored for subsequent proximate analysis.

### 2.7. Proximate Analysis, Cook Loss, and Warner–Bratzler Shear Force

Moisture and lipid content of loin chops and belly samples were determined using the chloroform–methanol solvent extraction method described by Novakofski et al. [29]. For belly samples, five 5 g subsamples of homogenized tissue were analyzed. For loin samples, 10 g portions of homogenized tissue (subcutaneous fat removed) were used. Moisture content was calculated from weight loss during drying, and lipid content was determined from weight loss following solvent extraction. Values are expressed as a percentage of initial sample weight.

Chops designated for Warner–Bratzler shear force (WBSF) analysis were aged for 14 d at 4 °C and subsequently stored at −20 °C until evaluation. WBSF and cook loss were determined according to the protocol described by Dilger et al. [30]. Briefly, chops were cooked on an open-hearth grill (Farberware model 455N; Walter Kidde, Bronx, NY, USA) to a final internal temperature of 63 °C, consistent with current recommendations for the safe preparation of whole-muscle pork in the United States. Internal temperature was monitored using a data-logging thermometer (Omega HH378; Omega Engineering, Norwalk, CT, USA). Weights were collected before and after cooking to determine cook loss. Following cooking and cooling, four 1.25 cm-diameter cores were removed from each chop parallel to the muscle fiber orientation. Cores were sheared perpendicular to the fiber direction using a Texture Analyzer (TA.HD Plus; Texture Technologies Corp., Scarsdale, NY, USA/Stable Micro Systems, Godalming, UK) equipped with a Warner–Bratzler blade, a 100 kg load cell, and a crosshead speed of 3.33 mm/s. Shear force values from the four cores were averaged to obtain a single value per chop.

### 2.8. Statistical Analysis

Data were analyzed using the MIXED procedure of SAS 9.4 (SAS Institute Inc., Cary, NC, USA). Each individual pig (n = 44) was considered the experimental unit, as genotype was inherent to the animal. The initial model included fixed effects of paternal IGF2 allele (A^PAT^ vs. G^PAT^), MSTN status (WT vs. HET), sex (barrow vs. gilt), and all corresponding interactions. Sex and its interactions with genotype were not significant (*p* > 0.05) and were therefore removed from the final model. The reduced model included fixed effects of paternal IGF2 allele, MSTN status, and their interaction. Least-squares means were generated, and pairwise comparisons were conducted using the PDIFF option in SAS. Effects were considered significant at *p* ≤ 0.05.

## 3. Results

### 3.1. Carcass Characteristics

Table 1 summarizes the main effects of MSTN status and paternal IGF2 genotype on carcass characteristics. Neither the main effect of the IGF2 genotype nor the MSTN × IGF2 interaction influenced carcass traits (*p* ≥ 0.15). Myostatin heterozygosity did not affect ending live weight (ELW) or hot carcass weight (HCW) (*p* ≥ 0.59). However, MSTN HET carcasses exhibited a greater dressing percentage compared with WT carcasses (*p* < 0.01). Fat-free lean percentage also increased (*p* < 0.01) by approximately 4 percentage units in HET carcasses relative to WT, primarily reflecting a greater loin muscle area (>10 cm^2^ increase; *p* < 0.01) and a numerically lower 10th-rib backfat depth (0.3 cm reduction; *p* = 0.11).

### 3.2. Primal Cut Weights

**Figure 1:**
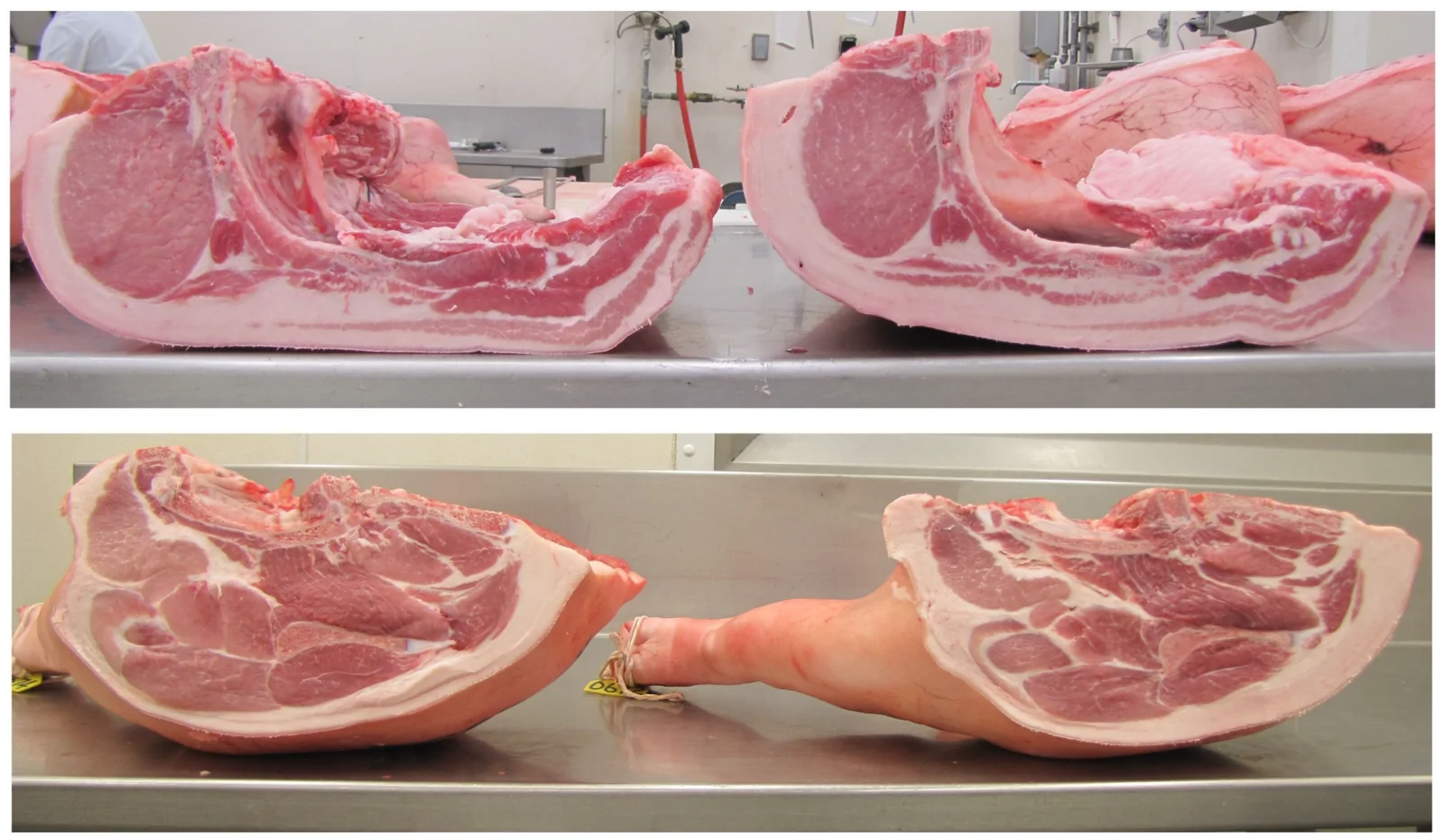
Representative loin/belly (**top**) and shoulder (**bottom**) primals from myostatin-heterozygous (MSTN HET; **left**) and wild-type (WT; **right**) pigs. MSTN HET pigs exhibit greater muscle area and reduced visible fat compared with WT pigs.

Table 2 summarizes the main effects of MSTN status and the paternal IGF2 allele on whole and trimmed primal weights. The MSTN × IGF2 interaction was not significant (*p* ≥ 0.14) for any primal weight, whether on an absolute basis or when expressed as a percentage of chilled side weight. Myostatin heterozygosity did not affect (*p* ≥ 0.24) Boston butt weights. However, whole and trimmed picnic weights, expressed as a percentage of chilled side weight, were greater (*p* ≤ 0.03) in HET carcasses compared with WT. Trimmed loin weight relative to chilled side weight was increased (*p* ≤ 0.01) in HET carcasses, whereas other loin weight measures did not differ (*p* ≥ 0.15). Ham weights, including whole and trimmed ham on both absolute and percentage bases, were greater (*p* ≤ 0.05) in HET compared with WT carcasses. In contrast, when expressed as a percentage of chilled side weight, both whole and trimmed bellies were lighter (*p* < 0.01) in HET compared with WT carcasses. These differences in primal weight and composition are visually illustrated in Figure 1.

The paternal IGF2 genotype did not influence (*p* ≥ 0.19) picnic weight or whole Boston butt weight. However, trimmed Boston butt weight, both absolute and relative to chilled side weight, was greater (*p* ≤ 0.03) in IGF2 A^PAT^ compared with IGF2 G^PAT^ carcasses. Whole loin weight was not affected by IGF2 genotype (*p* ≥ 0.28), but trimmed loin weight, both absolute and percentage-based, was increased (*p* ≤ 0.05) in A^PAT^ carcasses. Whole ham weight tended to be greater (*p* = 0.08), and trimmed ham weight (absolute and relative) was increased (*p* ≤ 0.01), in A^PAT^ compared with G^PAT^ carcasses. Absolute whole and trimmed belly weights did not differ (*p* ≥ 0.58) by IGF2 genotype; however, when expressed as a percentage of chilled side weight, whole and trimmed belly weights were reduced (*p* ≤ 0.03) in A^PAT^ carcasses.

### 3.3. Muscle Weights

Table 3 presents the main effects of MSTN status and paternal IGF2 genotype on individual muscle weights. A significant MSTN × IGF2 interaction was detected (*p* = 0.05) for semitendinosus weight, expressed as a percentage of chilled side weight (data not presented on tables). The semitendinosus percentage was greater (*p* ≤ 0.05) in HET A^PAT^ carcasses compared with all other genotype combinations. Absolute semitendinosus weight and all other muscle weights were not affected (*p* ≥ 0.17) by the interaction. However, both LTL and psoas major weights, expressed on an absolute basis and relative to chilled side weight, were greater (*p* ≤ 0.01) in HET compared with WT carcasses. Similarly, these muscles were heavier (*p* ≤ 0.04) in IGF2 A^PAT^ compared with G^PAT^ carcasses.

### 3.4. Loin Quality

Table 4 presents the main effects of MSTN status and paternal IGF2 genotype on loin quality traits. A significant MSTN × IGF2 interaction (*p* = 0.03) was detected for drip loss (interaction means not displayed). Drip loss was greater (*p* ≤ 0.05) in HET A^PAT^ loins compared with all other genotype combinations, which were not different from each other. Ultimate pH was reduced by 0.06 units in HET loins compared with WT. NPPC color score was decreased by 0.6 units (*p* < 0.01) in HET loins. Instrumentally, HET loins were lighter (L* increased by approximately 5 units; *p* < 0.01), less red (lower a*; *p* = 0.03), and more yellow (higher b*; *p* = 0.03) than WT loins. Loins from HET carcasses tended to have lower marbling scores (*p* = 0.06) and exhibited reduced lipid content (0.75 percentage units lower; *p* < 0.01) compared with WT loins. NPPC firmness scores and Warner–Bratzler shear force (WBSF) were not affected (*p* ≥ 0.15) by MSTN status. Paternal IGF2 genotype did not influence (*p* ≥ 0.16) any loin quality trait.

### 3.5. Belly Quality

Table 5 summarizes the effects of MSTN status and paternal IGF2 allele on belly quality traits. The MSTN × IGF2 interaction was not significant (*p* ≥ 0.18) for any belly measurement. Belly length and width were not affected (*p* ≥ 0.26) by MSTN status; however, belly thickness was reduced (*p* = 0.04) by more than 2 cm in HET compared with WT bellies. Belly flop distance was reduced by approximately 25% (*p* < 0.01), and lipid content was decreased by 9 percentage units (*p* < 0.01) in HET bellies relative to WT bellies. For the paternal IGF2 genotype, belly width tended to be greater in A^PAT^ carcasses, whereas length and thickness did not differ between genotypes. Belly flop distance was reduced by approximately 30% (*p* < 0.01), and lipid content was decreased by 4 percentage units (*p* < 0.01) in A^PAT^ compared with G^PAT^ bellies.

## 4. Discussion

The targeted *MSTN* loss-of-function allele evaluated in this study was introduced into germplasm segregating for the IGF2-G3072A variant to determine whether increased IGF2 expression would influence the phenotypic consequences of reduced MSTN activity. We hypothesized that the enhanced muscularity associated with the paternal IGF2 A^PAT^ allele might attenuate or mask the effects of partial MSTN deficiency, resulting in a greater phenotypic contrast between HET and WT pigs within the IGF2 G^PAT^ background. Contrary to this expectation, few significant MSTN × IGF2 interactions were detected for carcass composition or primal yields. Drip loss was the only trait exhibiting a significant MSTN × IGF2 interaction. Because no comparable interactions were detected for pH, color, marbling, tenderness, or carcass composition, it is unclear whether this isolated response reflects a biologically meaningful interaction between these loci or the greater sensitivity of water-holding capacity to subtle genetic effects. Additional studies with larger populations will be required to determine whether this interaction is reproducible. With the exception of drip loss, the effects of heterozygous MSTN loss of function were largely consistent across IGF2 genotypes. These findings indicate that, in the heterozygous state, reduced myostatin activity exerts effects on lean deposition that are primarily additive relative to the established effects of the IGF2 A^PAT^ allele. Although the modest sample size within each genotype combination may have limited statistical power to detect subtle interaction effects, the consistent direction and magnitude of the MSTN response across both IGF2 backgrounds support the conclusion that partial myostatin deficiency largely acts independently of IGF2 genotype.

Across livestock species, complete loss of MSTN function consistently results in increased muscularity, establishing myostatin as a conserved negative regulator of skeletal muscle growth. This phenotype has been documented in both naturally occurring and engineered mutations in sheep and cattle, including the well-characterized Texel sheep mutation and double-muscled cattle [3,4,31]. Studies of heterozygous cattle similarly demonstrate increased muscularity, with several breeds also exhibiting reduced adiposity, although the magnitude of fat-related responses appears to vary among genetic backgrounds [2,9,10,32]. Together, these studies support a dose-dependent response to reduced MSTN activity, with heterozygous cattle exhibiting an intermediate phenotype and complete loss-of-function animals demonstrating approximately two- to three-fold greater increases in muscularity [2,10]. In the present study, heterozygous MSTN pigs exhibited greater loin muscle area and increased fat-free lean percentage without differences in overall carcass weight. The increased yield of trimmed primals, particularly when expressed relative to chilled side weight, indicates enhanced lean accretion rather than increased carcass size. Comparable changes in carcass composition have also been reported in heterozygous MSTN pigs [11], supporting the conclusion that partial MSTN deficiency consistently shifts tissue partitioning toward lean deposition. The greater improvement in trimmed compared with whole primal yields, along with the reduction in extractable fat in the belly primal, suggests that increased cutability was driven not only by increased muscle deposition but also by reduced adipose tissue accretion. Collectively, these data demonstrate that partial MSTN loss of function shifts tissue partitioning toward lean deposition without increasing overall carcass mass.

Despite improvements in lean yield, heterozygous MSTN pigs exhibited reductions in several measures of loin quality. Both subjective and instrumental color assessments indicated that loins from HET pigs were lighter in color, accompanied by increased drip loss. Reduced water-holding capacity likely contributed to the paler appearance of the HET loins. Water loss from muscle decreases light absorption and increases surface reflectance, resulting in elevated L* values and a lighter visual appearance. This is of concern as color and purge loss strongly influence consumer purchasing decisions [33,34].

Decreased marbling scores and lower extractable lipid content further reflect the shift toward lean tissue deposition. Similar reductions in intramuscular fat have been reported in cattle carrying heterozygous MSTN mutations [2,35], suggesting that reduced marbling is a consistent consequence of diminished myostatin activity. In contrast, postmortem quality traits such as tenderness have been considerably less consistent among studies. In the present study, WBSF did not differ between genotypes. Although improved tenderness has been reported in some heterozygous and homozygous MSTN mutant cattle [8], and associations between MSTN haplotypes and tenderness have been described in sheep [36,37], many studies have reported little or no effect of MSTN mutations on overall meat quality or tenderness [2,9,38,39,40]. Collectively, these findings suggest that the most consistent meat quality consequences of partial MSTN deficiency are those associated with reduced fat deposition, altered appearance, and altered water-holding capacity, whereas effects on tenderness appear to depend more on species, genetic background, or the extent of myostatin inhibition.

Belly quality traits were similarly influenced by altered adipose deposition. HET bellies were thinner and less firm, as indicated by a reduced belly flop distance. Previous studies in MSTN loss-of-function pigs have similarly reported reduced carcass fat and smaller subcutaneous adipocytes [11,41], while heterozygous MSTN pigs have been shown to deposit a greater proportion of unsaturated fatty acids [42]. Although adipocyte size and fatty acid composition were not evaluated in the present study, these findings provide plausible mechanisms by which reduced myostatin activity may contribute to thinner, softer bellies. Similar directional effects were observed in pigs inheriting the IGF2 A^PAT^ allele in both the present study and previously [17], reinforcing that distinct genetic mechanisms promoting lean tissue accretion can produce comparable effects on pork belly quality.

From a commercial perspective, these findings highlight a tradeoff between lean yield and quality attributes important for further processing. Reduced belly firmness may impair bacon slicing performance, and increased purge loss may negatively influence retail appeal. Thus, while partial MSTN loss of function enhances lean deposition, the associated reduction in fat deposition presents a potential limitation in markets where belly quality and water-holding capacity are economically critical.

Although few significant interactions were detected in heterozygous animals, these findings should not be interpreted as evidence that genetic background is unimportant for MSTN function. Recent studies in modern commercial pig populations have similarly reported reduced survival of homozygous MSTN loss-of-function pigs, including fewer than expected homozygous offspring [43] and increased postnatal mortality or hindlimb weakness [43,44]. In contrast, complete MSTN deficiency has been successfully established in Chinese indigenous breed pigs without apparent survivability issues [14,15]. Collectively, these observations suggest that the consequences of complete MSTN deficiency may depend on genetic background. Whether this reflects interactions with major loci such as IGF2, cumulative effects of long-term selection for lean growth, or other modifier genes remains unknown and warrants further investigation.

## 5. Conclusions

Collectively, these findings demonstrate that partial loss of function of MSTN in heterozygous pigs shifts carcass composition toward increased lean deposition without increasing overall carcass weight. Improvements in loin muscle area, fat-free lean percentage, and trimmed primal yields reflect enhanced lean accretion rather than increased carcass size. Importantly, these effects were largely consistent across paternal IGF2 genotypes, indicating that in the heterozygous state, reduced myostatin activity acts primarily additively relative to the established lean-enhancing effects of the IGF2 A^PAT^ allele.

The increased lean yield observed in HET pigs was accompanied by reductions in several quality traits, including lighter loin color, increased drip loss, reduced intramuscular lipid content, and decreased belly firmness due to lower adipose deposition. These changes reflect a broader shift in tissue partitioning toward muscle and away from fat. While instrumental tenderness was not compromised, reductions in water-holding capacity and belly firmness may influence processing performance and consumer acceptance depending on market priorities. The commercial significance of these tradeoffs is therefore context-dependent. In production systems emphasizing lean yield and efficiency, partial MSTN loss of function may offer advantages. In contrast, markets where belly quality and bacon slicing performance are economically critical may be less tolerant of reduced fat deposition.

Beyond immediate production implications, the lethality associated with complete MSTN deficiency in commercial genetic backgrounds underscores an important biological consideration. The largely additive effects observed in heterozygotes suggest that a moderate reduction in myostatin activity is compatible with modern lean-selected pigs; however, the intolerance of homozygous loss of function highlights a need for further investigation into how long-term selection has shaped the developmental and metabolic constraints governing skeletal muscle growth. Understanding these system-level interactions will be essential for evaluating the future role of targeted growth-modifying alleles in commercial swine populations.

## Figures and Tables

**Table 1:** Effect of MSTN and IGF2 genotype on carcass characteristics.

	Genotype ^1^					*p*-Value		
	MSTN WT	MSTN HET	IGF2 G^PAT^	IGF2 A^PAT^	SEM	MSTN	IGF2	MSTN × IGF2
Pigs, n	24	20	23	21				
Ending live weight, kg	114.2	112.3	111.6	114.9	5.68	0.59	0.38	0.96
Hot carcass weight, kg	90.7	90.5	89.3	92.0	4.52	0.96	0.40	0.89
Dressing percentage, %	79.4	80.5	79.9	80.0	0.34	<0.01	0.80	0.51
Loin muscle area, cm^2^	52.0	62.7	56.6	58.1	1.45	<0.01	0.42	0.18
10th rib back fat depth, cm	2.12	1.82	1.96	1.97	0.20	0.11	0.99	0.66
Fat-free lean ^2^, %	53.9	57.8	56.0	55.7	1.17	<0.01	0.72	0.15

^1^ No MSTN LOF alleles (MSTN WT); one MSTN LOF allele (MSTN HET); paternally inherited IGF2 G allele (IGF2 G^PAT^); paternally inherited IGF2 A allele (IGF2 A^PAT^). ^2^ Fat-free lean was calculated as [(8.588 + (0.465 × HCW, lb) − (21.896 × fat thickness, in) + (3.005 × LMA, in^2^))/HCW, lb] × 100.

**Table 2:** Effect of *MSTN* and *IGF2* genotype on primal cut weights.

	Genotype ^1^					*p*-Value		
	MSTN WT	MSTN HET	IGF2 G^PAT^	IGF2 A^PAT^	SEM	MSTN	IGF2	MSTN × IGF2
Sides, n	24	20	23	21				
Boston butt, kg	4.37	4.35	4.25	4.46	0.365	0.90	0.26	0.77
% chilled side wt	10.22	10.13	10.14	10.21	0.384	0.71	0.79	0.76
Trimmed butt, kg	3.69	3.81	3.56	3.94	0.189	0.42	0.02	0.79
% chilled side wt	8.62	8.85	8.51	8.97	0.152	0.24	0.03	0.83
Picnic, kg	4.63	4.82	4.64	4.82	0.207	0.31	0.33	0.76
% chilled side wt	10.84	11.23	11.10	10.97	0.209	0.03	0.48	0.63
Trimmed picnic, kg	4.12	4.41	4.15	4.38	0.142	0.09	0.19	0.62
% chilled side wt	9.64	10.28	9.94	9.98	0.316	<0.01	0.80	0.27
Whole loin, kg	11.94	11.69	11.55	12.08	0.915	0.61	0.28	0.74
% chilled side wt	27.94	27.27	27.42	27.64	0.718	0.29	0.67	0.86
Trimmed loin, kg	9.55	10.13	9.44	10.24	0.444	0.15	0.05	0.63
% chilled side wt	22.37	23.49	22.60	23.27	0.294	<0.01	0.05	0.43
Whole ham, kg	10.68	11.41	10.71	11.38	0.450	0.05	0.08	0.89
% chilled side wt	25.04	26.60	25.56	26.07	0.477	<0.01	0.26	0.86
Trimmed ham, kg	9.17	10.24	9.22	10.18	0.258	<0.01	<0.01	0.32
% chilled side wt	21.60	24.02	22.11	23.50	1.052	<0.01	<0.01	0.21
Whole belly, kg	7.58	7.07	7.25	7.41	0.280	0.08	0.58	0.59
% chilled side wt	17.70	16.50	17.37	16.83	0.343	<0.01	0.03	0.14
Trimmed belly, kg	6.14	5.67	5.91	5.90	0.285	0.06	0.95	0.58
% chilled side wt	14.31	13.25	14.15	13.41	0.203	<0.01	<0.01	0.23

^1.^ No MSTN LOF alleles (MSTN WT); one MSTN LOF allele (MSTN HET); paternally inherited IGF2 G allele (IGF2 G^PAT^); paternally inherited IGF2 A allele (IGF2 A^PAT^).

**Table 3:** Effects of MSTN and IGF2 genotype on muscle weights.

	Genotype ^1^					*p*-Value		
Item	MSTN WT	MSTN HET	IGF2 G^PAT^	IGF2 A^PAT^	SEM	MSTN	IGF2	MSTN × IGF2
Muscles, n	24	20	23	21				
Longissimus dorsi, kg	3.16	3.76	3.28	3.64	0.092	<0.01	<0.01	0.80
% chilled side wt	7.42	8.80	7.88	8.34	0.320	<0.01	<0.01	0.19
Psoas major, kg	0.43	0.49	0.44	0.48	0.015	<0.01	0.03	0.82
% chilled side wt	1.01	1.17	1.05	1.13	0.065	<0.01	0.04	0.92
Semitendinosus, kg	0.57	0.60	0.56	0.61	0.283	0.17	0.02	0.17
% chilled side wt	1.34	1.40	1.33	1.41	0.073	0.05	0.03	0.05

^1.^ No MSTN LOF alleles (MSTN WT); one MSTN LOF allele (MSTN HET); paternally inherited IGF2 G allele (IGF2 G^PAT^); paternally inherited IGF2 A allele (IGF2 A^PAT^).

**Table 4:** Effects of MSTN and IGF2 genotype on loin quality characteristics.

	Genotype ^1^					*p*-Value		
Item	MSTN WT	MSTN HET	IGF2 G^PAT^	IGF2 A^PAT^	SEM	MSTN	IGF2	MSTN × IGF2
Loins, n	24	20	23	21				
Subjective Evaluation ^2^								
Color	3.0	2.4	2.8	2.6	0.28	<0.01	0.54	0.23
Marbling	1.6	1.3	1.5	1.3	0.14	0.06	0.23	0.85
Firmness	2.0	2.0	2.1	2.0	0.26	0.96	0.60	0.34
Instrumental Color ^3^								
L*	48.85	53.88	50.67	52.06	1.494	<0.01	0.22	0.67
a*	8.25	7.40	7.91	7.74	0.285	0.03	0.63	0.35
b*	2.63	3.50	2.92	3.21	0.547	0.03	0.46	0.37
Ultimate pH	5.63	5.57	5.60	5.60	0.057	0.04	0.89	0.32
Drip loss, %	3.65	6.00	4.97	4.68	0.626	<0.01	0.51	0.03
Moisture, %	74.39	75.10	74.76	74.73	0.603	<0.01	0.88	0.38
Lipid, %	2.50	1.75	2.05	2.20	0.234	<0.01	0.44	0.85
WBSF ^4^, kg	2.81	2.59	2.80	2.59	0.113	0.15	0.16	0.30

^1^ No *MSTN* LOF alleles (MSTN WT); one *MSTN* LOF allele (MSTN HET); paternally inherited IGF2 G allele (IGF2 G^PAT^); paternally inherited IGF2 A allele (IGF2 A^PAT^). ^2^ Color measured in half-point increments where 1 = palest, 6 = darkest; marbling measured in half-point increments based on estimated lipid content; firmness measured in half-point increments where 1 = softest, 5 = firmest. ^3^ Measured with Minolta colorimeter where greater L* indicates a lighter color, greater a* indicates a redder color, and greater b* indicates a more yellow color. ^4^ WBSF = Warner–Bratzler shear force.

**Table 5:** Effect of *MSTN* and *IGF2* genotype on belly quality characteristics.

	Genotype ^1^					*p*-Value		
Item	MSTN WT	MSTN HET	IGF2 G^PAT^	IGF2 A^PAT^	SEM	MSTN	IGF2	MSTN × IGF2
Bellies, n	24	20	23	21				
Length, cm	63.11	61.41	62.17	62.34	1.417	0.26	0.91	0.18
Width, cm	25.34	25.04	24.85	25.54	0.448	0.45	0.09	0.22
Thickness ^2^, mm	39.95	37.14	39.07	38.03	1.027	0.04	0.44	0.73
Flop Distance, cm	21.24	15.55	21.66	15.13	2.123	<0.01	<0.01	0.82
Moisture, %	52.16	59.03	53.99	57.21	2.094	<0.01	<0.01	0.88
Lipid, %	34.04	25.04	31.53	27.55	2.228	<0.01	<0.01	0.90

^1^ No *MSTN* LOF alleles (MSTN WT); one *MSTN* LOF allele (MSTN HET); paternally inherited IGF2 G allele (IGF2 G^PAT^); paternally inherited IGF2 A allele (IGF2 A^PAT^). ^2^ Thickness was an average of measurements from 8 locations from the anterior to posterior.

## Data Availability

All the data generated or analyzed during the present study are available from the author upon reasonable request.

## Abbreviations

The following abbreviations are used in this manuscript:

A^PAT^	paternally inherited IGF2 A allele
ELW	ending live weight
FFL	fat-free lean
G^PAT^	paternally inherited IGF2 G allele
HCW	hot carcass weight
HET	heterozygous
IGF2	insulin-like growth factor 2
INAD	investigational new animal drug
LMA	loin muscle area
LOF	loss of function
LTL	longissimus thoracis et lumborum
MSTN	myostatin
NPPC	National Pork Producers Council
WBSF	Warner–Bratzler shear force
WT	wild-type
